# Awareness of Hepatitis B Virus (HBV) Screening Before Marriage and Pregnancy Among Adults in the Al-Baha Region, Saudi Arabia

**DOI:** 10.7759/cureus.52057

**Published:** 2024-01-10

**Authors:** Ramy H Agwa, Taher H Elwan, Hashim Abdulrahman S Alghamdi, Abdullah Ali S Alghamdi, Fatema Ibrahim A Altaweel, Abdullah A Alghamdi, Hawraa A Alhussain, Khader Mohammed A Alsawlihah, Faisal A Alzahrani

**Affiliations:** 1 Internal Medicine/Hepatology and Gastroenterology, Mansoura University, Mansoura, EGY; 2 Internal Medicine, College of Medicine, Al-Baha University, Al-Baha, SAU; 3 General Surgery, College of Medicine, Al-Baha University, Al-Baha, SAU; 4 Medicine, College of Medicine, Al-Baha University, Al-Baha, SAU

**Keywords:** pregnancy, marriage, awareness, screening, hbv

## Abstract

Background

Hepatitis B is a global public health concern. Understanding the awareness, testing practices, and vaccination status of individuals is crucial for effective prevention and control strategies. This study aims to assess these aspects among participants in the Al-Baha region, Saudi Arabia.

Methodology

A cross-sectional study was conducted among 440 participants. Demographic data, awareness of hepatitis B, knowledge of transmission modes, symptoms, and complications were collected through a structured questionnaire. Participants' testing and screening practices, sources of information, and vaccination status were also assessed. Data were analyzed using descriptive statistics and associations were explored using chi-square tests.

Results

The majority of participants were females (51.8%) and aged 18-25 years (55.2%). While most participants had heard of hepatitis B (85.7%), only a small percentage correctly identified sexual contact as a mode of transmission (38.6%). Knowledge regarding symptoms and complications was moderate, with 52.3% correctly identifying symptoms and 69.8% recognizing liver damage and cirrhosis as complications. Although awareness of screening was high (84.8%), the actual practice was low (35.0%). Education was the least reported source of information, while the internet (46.7%) and health care provider (27.6%) were commonly mentioned. Approximately half of the participants reported receiving the hepatitis B vaccine (48.9%), but a significant proportion had not completed all vaccine doses (55.0%).

Conclusion

The study revealed moderate awareness of hepatitis B among the participants, but knowledge gaps existed regarding transmission modes and complete symptom recognition. Testing and screening practices were suboptimal, with low rates of screening despite high awareness. Vaccination uptake was moderate, but incomplete vaccine schedules were prevalent. Targeted educational campaigns are needed to address knowledge gaps, promote testing and completion of vaccination schedules, and enhance the role of healthcare providers in disseminating accurate information. Improving knowledge and practices related to hepatitis B can strengthen public health efforts, enhance prevention, and control strategies.

## Introduction

Hepatitis B virus (HBV) is a highly contagious liver infection that can lead to chronic liver disease, cirrhosis, and liver cancer. HBV spreads through blood and body fluids, such as semen, vaginal fluids, and breast milk [1]. In Saudi Arabia, HBV is a major public health problem. According to the World Health Organization (WHO), an estimated 2.5 million people in Saudi Arabia are living with chronic HBV infection [1].

The Centers for Disease Control and Prevention (CDC) recommends that all pregnant women should be screened for hepatitis B surface antigen (HBsAg) during each pregnancy, preferably in the first trimester. This is regardless of their vaccination status or previous test results. Women who have been previously screened for HBsAg and have not been exposed to the virus since then only need to be screened for HBsAg [2]. A number of studies have been conducted in Saudi Arabia to assess awareness about HBV screening. These studies have found that awareness about HBV screening is low, with rates ranging from 20% to 50% [3-5].

There are a number of reasons why awareness about HBV screening is low in Saudi Arabia. These reasons include: Lack of knowledge about HBV. Many people in Saudi Arabia are not aware of the risks of HBV infection or the benefits of HBV screening [4-6]. Fear of the test: Some people are afraid of getting tested for HBV because they fear that they may be infected [7]. Cost: The cost of HBV screening can be a barrier for some people [7]. The low awareness about HBV screening in Saudi Arabia is a major public health concern. HBV is a serious disease that can cause significant health problems. Early detection and treatment of HBV can help to prevent these health problems. Therefore, the aim of the current study was to assess the awareness of HBV screening before marriage and pregnancy among adults in the Al-Baha region in Saudi Arabia.

## Materials and methods

The study utilized a cross-sectional design to assess the knowledge and awareness about HBV screening before marriage and pregnancy in the Al-Baha region of Saudi Arabia. The study was conducted from June 21, 2023, to September 21, 2023.

Sampling procedure

The study included adults residing in the Al-Baha region who met the inclusion criteria. A sample size of 384 participants was deemed appropriate for ensuring generalizability.

Data collection and management

Data were collected using a questionnaire created by a gastroenterology consultant and his team in both paper and Google Forms formats. Data collectors distributed paper forms to participants at various locations, including Al-Baha Mall, Al-Ghonaim Mall, Al-Baha Boulevard, Raghdan Park, Al-Hossam Park, Al-Mekhwah Park, Saturday Public Market in Baljurashi, Al-Danah Park, and Al-Hawiah Walkway. Participants were informed about the study aims and assured of data confidentiality. Consents were obtained from participants at the beginning and before completing the questionnaire. All researchers and data collectors performed data entry, and after verification, the data were transferred to a statistical database.

Inclusion criteria

The inclusion criteria for participants were adults aged 18 years and above who agreed to participate in the study.

Exclusion criteria

Individuals below 18 years of age and those working in the medical field were excluded from the study.

Data analysis plan

Parametric approaches were used to describe numerical data, such as means, standard deviation (SD), or medians. Percentages were used to describe categorical variables. The Statistical Package for the Social Sciences (SPSS) version 26.0 (IBM Corp., Armonk, NY, USA) was employed for data entry, analysis, and presentation. An appropriate statistical test, such as the Student's t-test or Mann-Whitney test for numerical data and the chi-square test for categorical data, was used to compare groups. Statistical significance was considered at a p-value of less than 0.05.

Ethical considerations

Written or verbal consent was obtained from all participants, and data were kept securely. The research staff ensured confidentiality and privacy, and no private information was discussed during the study analysis.

## Results

Table 1 presents the demographic factors of the participants (N=440). The majority of the participants were females 255 (58.0%) while 185 (42.0%) were males. In terms of age, the largest age group was 18-25 years old, accounting for 249 (55.2%) of the participants. Regarding marital status, the majority of participants were single (244; 55.5%), followed by married individuals (187; 42.5%). In terms of educational level, the highest proportion had a bachelor's degree (291; 66.1%), followed by high school education (129; 29.3%), and a smaller percentage had a master's degree (16; 3.6%). The income level of the participants was distributed as follows: low income 91 (20.7%), medium income 289 (65.7%), and high income 60 (13.6%). In terms of residency, the majority of participants were from Al-Baha (261; 59.3%), followed by Baljurashi (43; 9.8%) and Al Quora (41; 9.3%).

**Table 1:** Demographic factors of the participants (N=440)

		Count	Column N %
Gender	Male	185	42.0%
	Female	255	58.0%
Age	18-25	243	55.2%
	26-35	82	18.6%
	36-45	59	13.4%
	46-55	51	11.6%
	> 55	5	1.1%
Marital status	Single	244	55.5%
	Married	187	42.5%
	Divorced	9	2.0%
Educational level	No certificates	1	0.2%
	Elementary	0	0.0%
	Middle school	3	0.7%
	High school	129	29.3%
	Bachelor’s	291	66.1%
	Master	16	3.6%
Income level	Low	91	20.7%
	Medium	289	65.7%
	High	60	13.6%
Residency	Al-Baha	261	59.3%
	Al Makhwah	24	5.5%
	Qilwah	5	1.1%
	Al Mandaq	19	4.3%
	Baljurashi	43	9.8%
	Ash Shu'ara	2	0.5%
	Beni Hassan	5	1.1%
	Al Quara	41	9.3%
	Ghamid Alzinad	18	4.1%
	Al Aqiq	22	5.0%

Table 2 provides information on the participants' awareness of HBV infection. The majority of participants had heard of hepatitis B (377; 85.7%). When asked about how HBV is spread from person to person, 170 of them (38.6%) provided the correct answer, which is through sexual contact, while the majority of participants (270; 61.4%) gave an incorrect response. Regarding symptoms associated with HBV infection, 230 of them (52.3%) knew the correct symptoms, including fever, fatigue, abdominal pain, and jaundice. When asked about the complications resulting from chronic HBV infection, 307 of them (69.8%) correctly identified liver damage and cirrhosis as potential complications. In terms of testing for hepatitis B, the majority of participants (266; 60.5%) reported that they had not been evaluated, while 174 of them (39.5%) had undergone testing. Among those who were evaluated, the most common testing location was a hospital (143; 82.2%), followed by a clinic in 16 participants (9.2%), 13 in a private laboratory (7.5%), and two in other locations (1.1%).

**Table 2:** Awareness of hepatitis B virus (HBV) infection

		Count	Column N %
Have you ever heard of hepatitis B?	No	63	14.3%
	Yes	377	85.7%
How is HBV spread from person to person?	Incorrect	270	61.4%
	Correct	170	38.6%
Are there any symptoms associated with HBV infection? If so, what are they?	Incorrect	210	47.7%
	Correct	230	52.3%
Which of the following complications can result from chronic HBV infection?	Incorrect	133	30.2%
	Correct	307	69.8%
Have you ever been evaluated for hepatitis B?	No	266	60.5%
	Yes	174	39.5%
If yes, where did you get tested?	Hospital	143	82.2%
	Clinic	16	9.2%
	Private laboratory	13	7.5%
	Other	2	1.1%

Table 3 provides insights into the awareness of HBV screening among the participants. The majority of participants (78.4%) had heard of hepatitis B screening, while 95 of them (21.6%) had not. Regarding the purpose of hepatitis B screening, 304 (69.1%) of participants knew its purpose and 176 (40.0%) of participants reported being screened for hepatitis B. Among those who were screened, the most common location was a hospital by 145 participants (82.4%), followed by a clinic in 18 participants (10.2%) and private laboratory in 12 participants (6.8%). The importance of hepatitis B screening before marriage was recognized by 410 (93.2%) participants and 392 (89.1%) participants acknowledged the importance of screening during pregnancy. When asked about the reasons for the importance of screening before marriage and during pregnancy, the majority of participants (248; 58.1%) indicated that it was to address all potential transmission routes, including mother-to-child transmission and transmission between partners. Additionally, 92 (21.5%) recognized the importance of screening to prevent mother-to-child transmission, 30 (7.0%) highlighted the importance of preventing transmission between partners, and 19 (4.4%) mentioned the significance of receiving appropriate medical care and treatment if infected. Among married participants, 72 (38.5%) had undergone HBV screening before marriage. Regarding pregnancy history, 72 (28.2%) of female participants had been pregnant at some point among female participants, 16 of them (22.0%) reported being screened for HBV, while 56 (78.0%) had not undergone screening. For those who were screened during pregnancy, 11 of them (68.7%) indicated that the screening was ordered by their obstetrician, while the other five (31.3%) performed the screening themselves (Table 3).

**Table 3:** Awareness of hepatitis B virus (HBV) screening

		Count	Column N %
Have you ever heard of hepatitis B screening?	No	95	21.6%
	Yes	345	78.4%
Do you know the purpose of hepatitis B screening?	No	136	30.9%
	Yes	304	69.1%
Have you ever been screened for hepatitis B?	No	264	60.0%
	Yes	176	40.0%
If yes, where did you get screened?	Hospital	145	82.4%
	Clinic	18	10.2%
	Private laboratory	12	6.8%
	Other	1	0.6%
Do you think it is important to be screened for hepatitis B before marriage?	No/ I do not know	30	6.8%
	Yes	410	93.2%
Do you think it is important to be screened for hepatitis B during pregnancy?	No/ I do not know	48	10.9%
	Yes	392	89.1%
If yes, why do you think it is important to be screened for hepatitis B before marriage and during pregnancy?	To prevent the transmission of hepatitis B from mother to child	92	21.5%
	To prevent the transmission of hepatitis B from one partner to another	30	7.0%
	To ensure proper medical care and treatment if infected	19	4.4%
	All of the above	248	58.1%
	I do not know	38	8.9%
If you are married, have you screened for HBV before marriage?	No	115	61.5%
	Yes	72	38.5%
For females, have you been pregnant now or before?	No	183	71.8%
	Yes	72	28.2%
If the previous answer was yes, have you screened for HBV?	No	56	78.0%
	Yes	16	22.0%
If the previous answer was yes, how did you screen for HBV?	From myself	5	31.3%
	My obstetrician ordered it	11	68.7%

**Figure 1:**
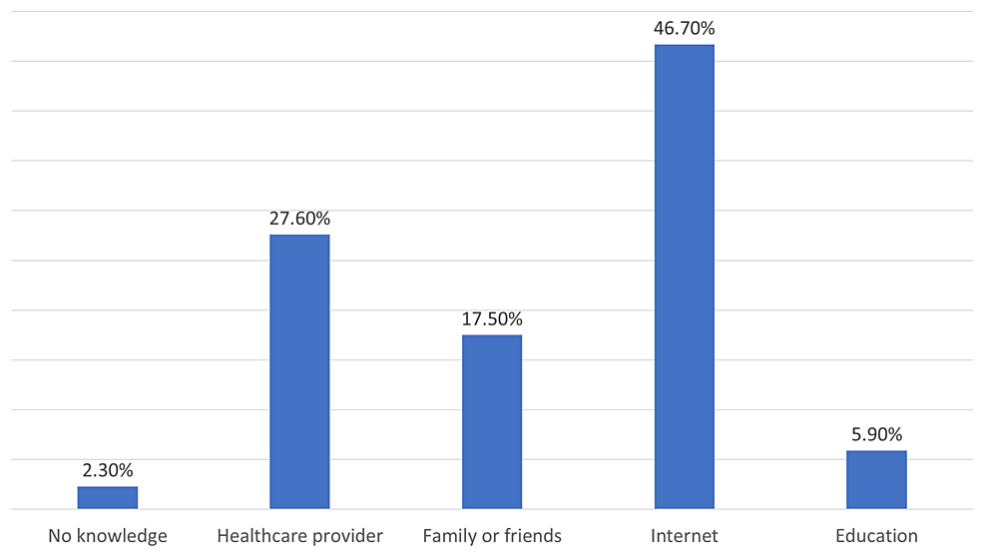
What are the sources of information you have about hepatitis B virus (HBV) and HBV screening?

Regarding sources of information, the internet, followed by healthcare providers, were mentioned by 46.7% and 27.6% of participants, respectively, family or friends by 17.5%, and education by 5.9%. A small proportion (2.3%) reported having no knowledge about HBV and HBV screening (Figure 1).

Table 4 provides information on the vaccination status of the participants. Approximately 50.5% of participants reported receiving the hepatitis B vaccine, and among those who received the vaccine, the majority (87.8%) received it at a hospital, while a smaller percentage (12.2%) received it at a clinic. Regarding the completion of all vaccine doses, 42.5% of participants had completed all doses. Reasons for not completing all doses included forgetting to schedule or missing appointments (17.4%), experiencing side effects or adverse reactions (3.2%), inability to afford the vaccine (4.7%), lack of awareness about the need to complete all doses (52.2%), and other reasons (22.5%).

**Table 4:** Vaccination status

		Count	Column N %
Have you ever received the hepatitis B vaccine?	No	218	49.5%
	Yes	222	50.5%
If yes, where did you receive the vaccine?	Hospital	195	87.8%
	Clinic	27	12.2%
	Private laboratory	0	0.0%
	Other	0	0.0%
Have you completed all doses of the hepatitis B vaccine?	No	253	57.5%
	Yes	187	42.5%
If no, why were you not able to complete all doses?	Forgot to schedule or missed appointments	44	17.4%
	Experienced side effects or adverse reactions	8	3.2%
	Could not afford the vaccine	12	4.7%
	Not aware of the need to complete all doses	132	52.2%
	Other	57	22.5%

**Figure 2:**
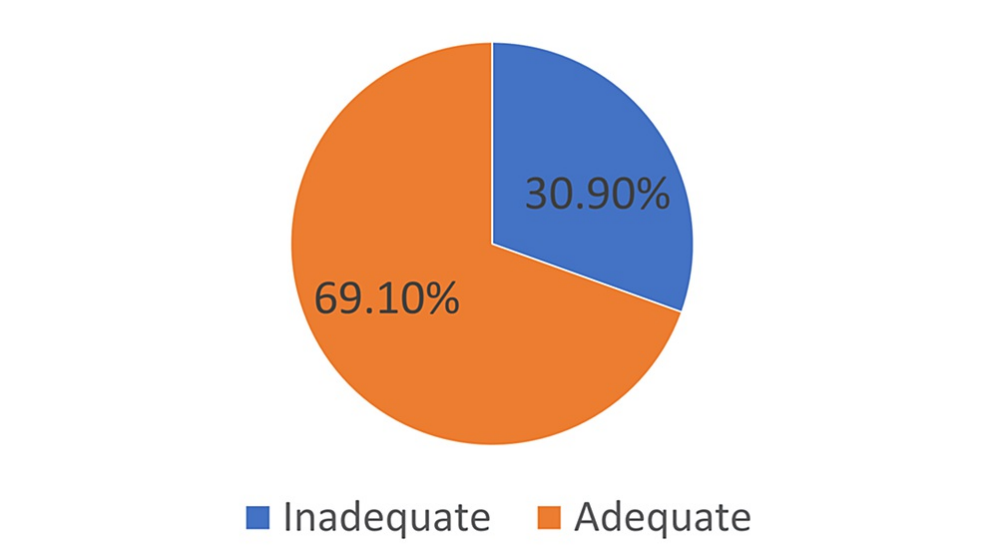
Distribution of the participants depending on their knowledge

The distribution of participants based on their knowledge about hepatitis B is presented in Figure 2. Approximately 69.1% of participants had adequate knowledge, while 30.9% had inadequate knowledge.

Table 5 examines the relationship between knowledge, demographic factors, and the practice of testing and vaccination. The analysis reveals that gender did not significantly influence knowledge (p-value = 0.182). Similarly, age, marital status, and income level did not show significant associations with knowledge. However, educational level demonstrated a significant association with knowledge (p-value = 0.081), with participants having a higher educational level more likely to possess adequate knowledge about hepatitis B. Regarding sources of information, participants who obtained information from healthcare providers were more likely to have adequate knowledge (p-value < 0.001). Participants who received information from family or friends, the internet, or education also demonstrated a higher likelihood of having adequate knowledge. The practice of testing and vaccination was strongly associated with knowledge. Participants with adequate knowledge were more likely to have been evaluated for hepatitis B (p-value < 0.001), screened for hepatitis B (p-value < 0.001), and received the hepatitis B vaccine (p-value < 0.001) compared to those with inadequate knowledge.

**Table 5:** The relation between knowledge and demographic factors and practice of testing and vaccination

		Knowledge				
		Inadequate		Adequate		P-value
		Count	Row N %	Count	Row N %	
Gender	Male	71	33.5%	141	66.5%	0.182
	Female	63	27.6%	165	72.4%	
Age	18-25	74	30.5%	169	69.5%	0.981
	26-35	24	29.3%	58	70.7%	
	36-45	19	32.2%	40	67.8%	
	46-55	16	31.4%	35	68.6%	
	> 55	1	20.0%	4	80.0%	
Marital status	Single	85	30.2%	196	69.8%	0.840
	Married	47	31.3%	103	68.7%	
	Divorced	2	22.2%	7	77.8%	
Educational level	No certificates	0	0.0%	1	100.0%	0.081
	Elementary	0	0.0%	0	0.0%	
	Middle school	3	100.0%	0	0.0%	
	High school	43	33.3%	86	66.7%	
	Bachelor’s	83	28.5%	208	71.5%	
	Master	5	31.3%	11	68.8%	
Income level	Low	33	36.3%	58	63.7%	0.393
	Medium	83	28.7%	206	71.3%	
	High	18	30.0%	42	70.0%	
What are the sources of information you have about HBV and HBV screening?	Healthcare provider	14	11.6%	107	88.4%	0.000*
	Family or friends	31	40.3%	46	59.7%	
	Internet	81	39.5%	124	60.5%	
	Education	0	0.0%	26	100.0%	
Have you ever been evaluated for hepatitis B?	No	115	39.8%	174	60.2%	0.000*
	Yes	19	12.6%	132	87.4%	
Have you ever been screened for hepatitis B?	No	115	40.2%	171	59.8%	0.000*
	Yes	19	12.3%	135	87.7%	
Have you ever received the hepatitis B vaccine?	No	99	44.0%	126	56.0%	0.000*
	Yes	35	16.3%	180	83.7%	

## Discussion

Hepatitis B is a matter of great importance in the field of public health on a global scale [8]. It is imperative to comprehend the level of knowledge, testing practices, and vaccination status among individuals in order to devise effective measures for prevention and management. The present research offers significant insights into these particular aspects among the general population in the Al-Baha region, Saudi Arabia.

The present study offers an examination of the participants' level of awareness regarding HBV infection. A significant proportion of the participants demonstrated familiarity with hepatitis B, suggesting a satisfactory degree of knowledge among the subjects involved in the study which is similar to the results of some previous studies [9,10]. Nevertheless, when queried regarding the methods of transmission, only a minority of respondents supplied the accurate response of sexual contact. The present results indicate a deficiency in understanding pertaining to the principal mode of transmission of hepatitis B. The findings presented in this study align with other research that has documented a lack of comprehensive understanding among the general populace regarding the various mechanisms through which hepatitis B can be transmitted [11-13]. Addressing these knowledge gaps is of utmost importance, necessitating the implementation of focused educational efforts aimed at promoting accurate information on the transmission of hepatitis B.

In relation to symptoms and complications, slightly more than 50% of the participants accurately recognized the symptoms linked to HBV infection. This suggests a moderate amount of understanding of the clinical presentations of the disease which was also reported in some previous studies [14,15]. Furthermore, a notable percentage of participants demonstrated awareness of the possible problems associated with persistent HBV infection, specifically liver damage and cirrhosis [16,17]. The aforementioned results are promising and indicate that individuals possess a certain level of comprehension regarding the enduring ramifications of hepatitis B. Nevertheless, it is imperative to pursue additional education to augment one's understanding of the complete spectrum of symptoms and difficulties linked to the ailment.

Furthermore, the present study elucidates the participants' cognizance and implementation of testing and screening protocols for hepatitis B. A significant proportion of the participants demonstrated familiarity with hepatitis B screening, suggesting a satisfactory degree of knowledge regarding this preventive intervention. Furthermore, a substantial fraction of individuals acknowledged the significance of undergoing screening prior to marriage and throughout pregnancy, a stance that is consistent with established protocols and expert suggestions [18,19]. Nevertheless, the implementation of screening measures was somewhat limited, as indicated by the fact that only 35.0% of the study participants reported undergoing screening for hepatitis B. A similar prevalence of screening was reported in different studies including 27.4% in France [11] and 12.2% in the Republic of Congo [20]. The aforementioned results indicate a disparity between knowledge and behavior, underscoring the necessity for focused interventions aimed at encouraging the adoption of hepatitis B screening. This is particularly crucial among high-risk demographics, such as persons intending to marry or women in the prenatal stage.

The survey participants indicated that they acquired knowledge from a range of sources, with healthcare providers being the least often cited source. The results are worrisome given the crucial role that healthcare personnel fulfill in the dissemination of precise information and the promotion of preventive measures. There is a need to prioritize initiatives aimed at strengthening the involvement of healthcare providers in the dissemination of knowledge pertaining to hepatitis B, encompassing its transmission, diagnostic tests, and immunization. The internet has been identified as a prominent and noteworthy source of information, underscoring the considerable impact of digital platforms in molding knowledge and fostering awareness [21]. Nevertheless, it is crucial to emphasize the need to guide users towards dependable sources due to the fluctuating nature of internet information in terms of its quality and accuracy [22]. The study also highlighted the inclusion of family and friends as valuable sources of knowledge, suggesting the possibility of utilizing peer education and support to promote awareness of hepatitis B.

The data revealed information regarding the participants' vaccination status. Roughly 50% of the individuals said that they had received the hepatitis B vaccine, suggesting a moderate level of vaccination adoption among the study cohort. In a previous study, the authors reported that 47% of the general population had been vaccinated against HBV [11], while another study reported a rate of 40.1% [12], and a study by Rajamoorthy et al. showed that only 26.4% had been vaccinated [23]. The data indicates that a significant proportion of vaccinated persons obtained their hepatitis B vaccinations from hospital settings, highlighting the crucial role that healthcare facilities play in the provision of such immunization services. Nevertheless, a significant majority of the participants did not successfully adhere to the recommended vaccination regimen, citing various factors such as forgetfulness, experiencing adverse effects, financial constraints, and a lack of understanding of the importance of completing the whole course of vaccination. It is imperative to address the obstacles hindering the completion of the vaccine schedule by the implementation of focused interventions [24,25]. These interventions should encompass reminder systems, comprehensive information regarding potential adverse effects, and measures to guarantee the affordability and accessibility of vaccines.

The analysis of participant distribution according to their knowledge of hepatitis B indicates that a significant portion of participants possessed sufficient information. This finding is indicative of a positive trend, as those who possessed sufficient knowledge showed a higher propensity to partake in testing and immunization behaviors [13,26-29]. The findings of the research conducted in the present study provide additional support for this conclusion, demonstrating a positive association between educational attainment and knowledge levels.

Although the study offers interesting insights, it is important to recognize several limitations. It is possible that the recruitment approach employed in the study or the specific area where the study was conducted may have created a selection bias, hence potentially influencing the demographic characteristics of the participants. Additionally, the research was dependent on data obtained through self-reporting, a method that is susceptible to biases related to recollection and social desirability. The responses of the participants might have been subject to social desirability bias, perhaps leading to a distortion of their true knowledge, habits, and views. Ultimately, due to the study's limited scope within a particular geographic area, it is important to acknowledge that the findings may not be generalizable to the full populace of the nation or other geographical regions.

## Conclusions

This study brings attention to significant factors pertaining to the knowledge, testing behaviors, and immunization status of persons in relation to hepatitis B. Although there was a moderate level of awareness regarding hepatitis B, deficiencies in information were observed pertaining to the mechanisms of transmission and comprehensive recognition of symptoms. The investigation additionally unveiled a disparity between knowledge and practical implementation, characterized by limited rates of testing and insufficient adherence to immunization schedules. Significant sources of information were determined to include healthcare providers, as well as the internet and peer networks. It is recommended that targeted educational programs should be designed with a specific focus on highlighting the significance of testing and completing the vaccine series. These campaigns should aim to address the identified gaps in knowledge and encourage the adoption of preventative behaviors. Enhancing awareness and competence in the realm of hepatitis B, together rectifying these deficiencies, can bolster public health initiatives, ultimately resulting in enhanced disease prevention and control.

## Appendices

**Table 6:** Questionnaire

S.No.	Question	Response	Code
1	Age	18 years old	
		18 – 25 years old	
		26 – 35 years old	
		36 – 45 years old	
		46 – 55 years old	
		> 55 years old	
2	Gender	Male	
		Female	
3	Marital status	Single	
		Married	
		Divorced	
4	Education level	No certificate.	
		Elementary school.	
		Middle school.	
		High school.	
		Bachelor’s	
		Master.	
		Other …….	
5	Occupation	(Optinal)	
6	Socioeconomic status	Low.	
		Middle,	
		High	
7	Place of residence	Al-Baha	
		Al Makhwah	
		Qilwah	
		Al Mandaq	
		Baljurashi	
		Ash Shu'ara	
		Beni Hassan	
		Al Quara	
		Ghamun Alzinad	
		Al Aqiq	

8	Have you ever heard of hepatitis B?	Yes.
		No.
9	How is HBV spread from person to person?	Through mosquito bites.
		Through contaminated food and water.
		Through sexual contact.
		All of the above.
		None of the above.
		I don’t know.
10	Are there any symptoms associated with HBV infection? If so, what are they?	No, there are no symptoms.
		Yes, symptoms include fever, fatigue, and abdominal pain.
		Yes, symptoms include yellowing of the skin and eyes (jaundice).
		Yes, symptoms include joint pain and rash.
		I don't know.
11	Which of the following complications can result from chronic HBV infection?	Liver damage and cirrhosis
		Kidney failure
		Heart disease
		Lung cancer
		None of the above
		I don't know.
12	Have you ever been tested for hepatitis B?	Yes
		No
13	If yes, where did you get tested?	Hospital
		Clinic
		Private laboratory
		Other (please specify)
14	Have you ever heard of hepatitis B screening?	Yes.
		No.
15	Do you know the purpose of hepatitis B screening?	Yes.
		No.
16	Have you ever been screened for hepatitis B?	Yes.
		No.
17	If yes, where did you get screened?	Hospital
		Clinic
		Private laboratory
		Other (please specify)
18	Do you think it is important to be screened for hepatitis B before marriage?	Yes.
		No.
		I don’t know.
19	Do you think it is important to be screened for hepatitis B during pregnancy?	Yes
		No
		I don’t know
20	If yes, why do you think it is important to be screened for hepatitis B before marriage and during pregnancy?	To prevent the transmission of hepatitis B from mother to child
		To prevent the transmission of hepatitis B from one partner to another
		To ensure proper medical care and treatment if infected
		All of the above
		I don't know
21	If you’re married, have you screened for HBV before marriage?	Yes
		No
		I’m not married.
22	For female, have being pregnant now or before?	Yes
		No
		I’m male.
23	If the previous answer was yes, have you screened for HBV?	Yes
		No
		The previous answer was no or I’m male.
24	If the previous answer was yes, how did you screen for HBV?	From myself.
		My obstetrician ordered it.
		The previous answer was no or I’m male.
25	What are the sources of information you have about HBV and HBV screening?	Healthcare provider
		Family or friends
		Internet
		Other (please specify)
26	Have you ever received the hepatitis B vaccine?	Yes
		No
27	If yes, where did you receive the vaccine?	Hospital
		Clinic
		Private healthcare provider
		Other (please specify)
28	Have you completed all doses of the hepatitis B vaccine?	Yes
		No
29	If no, why were you not able to complete all doses?	Forgot to schedule or missed appointments.
		Experienced side effects or adverse reactions
		Couldn't afford the vaccine.
		Not aware of the need to complete all doses.
		Other reasons (please specify)
		I have completed all doses of the hepatitis B vaccine
